# The double opioid crisis: A call for balance

**DOI:** 10.1002/pds.4720

**Published:** 2019-01-17

**Authors:** Marjolein J.M. Vranken, Marie‐Hélène D.B. Schutjens, Aukje K. Mantel‐Teeuwisse

**Affiliations:** ^1^ Utrecht Institute for Pharmaceutical Sciences (UIPS), Division of Pharmacoepidemiology and Clinical Pharmacology Utrecht University Utrecht the Netherlands; ^2^ Schutjens de Bruin Tilburg the Netherlands

**KEY POINTS**

- The opioid crisis can be seen as a double crisis or a dual epidemic: the crisis of nonmedical use and the crisis of uncontrolled pain.
- It is important to ensure both crisis are adequately addressed.
- The evidence base of clinical guidelines to ensure safe and appropriate opioid prescribing as well as compliance with  these  guidelines  can  be  further  improved.
- There is a striking absence on research related to the lack of equal access to medically justified opioids. Additionally, there is a lack of evidence on unintended consequences of policy and regulatory actions.
- Strategies to battle nonmedical use of opioids should not go at the expense of access for patients in legitimate medical need. Close monitoring is needed to minimize unintended consequences.

## 1 INTRODUCTION

In this issue of *Pharmacoepidemiology and Drug Safety,* several research papers address the safe and appropriate use of opioids. This is important, given the critical situation on opioid use in the United States (US). According to preliminary data from the Centers for Disease Control and Prevention (CDC), more than 72 000 people in the United States died because of drug overdose in 2017 with over two‐thirds involving opioids.^1^ This situation has been declared a “national public health emergency.”^2^ To address the so‐called opioid epidemic, the House and Senate recently passed new legislation that aims to increase access to opioid dependence treatment and to prevent illicit opioids from entering the market, and supports research on other treatment options for pain besides opioids.^3^, ^4^ Experts and activists are skeptical whether this agreement will solve the opioid crisis, at least in part because of limited funding.^3^

## 2 THE DOUBLE CRISIS: NONMEDICAL USE AND UNCONTROLLED PAIN

We acknowledge that abuse and diversion of opioids constitute a serious threat to public health. But it is also important to recognize that there is another side of the coin: opioids are an indispensable pharmaceutical treatment option for patients in pain, and many patients in medical need have inadequate access. Data from the International Narcotics Control Board (INCB) show that 95.7% of the global consumption of opioid analgesics in 2011 to 2013 took place in regions representing only 15% of the global population.^5^ In the light of these observed inequities, the opioid crisis can also be seen as two separate crises or a dual epidemic, both requiring our attention: the crisis of nonmedical use of opioids and the crisis of uncontrolled pain.^6^ The latter—the undertreatment of pain—is not a new problem originating from the nonmedical use crisis, but a long‐standing problem for a major part of the global population. Although both crises are important, in discussions, the nonmedical use of opioids tends to be prioritized over the undertreatment of pain. How can we ensure that both are adequately addressed, and in particular, how can we prevent collateral damage in the crisis of uncontrolled pain?

## 3 CLINICAL GUIDELINES TO SUPPORT THE SAFE AND APPROPRIATE USE OF OPIOIDS

In recent years, there has been a large increase in the number of scientific studies reporting on the nonmedical use of opioids. Despite this scientific focus, there is still a lack of evidence‐based guidance on the safe prescribing of opioid medicines and on the pathways from opioid dependence to opioid overdosing. Several studies have identified medication‐related and patient‐related factors associated with the risk of opioid overdose. Medication‐related factors include the use of long‐acting or extended release formulations (especially within the first 2 weeks of initiation of therapy), combined use with benzodiazepines, high daily doses, and long‐term opioid use.^7^ The latter two factors are also associated with the risk of opioid dependence.^7^ Patient‐related factors associated with opioid dependence include a history of substance use disorder. Most of these risk factors are reflected in the CDC guidelines on opioid prescribing for chronic noncancer pain, which were published^8^ in 2016. As opioid overdoses in the United States appear increasingly to be associated with heroin use (potentially mixed with illicit fentanyl) rather than the use of prescription opioids, there is also a high need for evidence on different pathways to opioid overdose, taking into account complex longitudinal patterns of prescribed and nonprescribed opioids.^9^

Prescribing guidelines are important to guide clinicians in the safe and appropriate prescribing of opioids. However, the CDC guidelines are also criticized for making some recommendations that are not supported by current scientific evidence, which is also acknowledged by the guidelines themselves.^8^, ^10^ Although the CDC guidelines categorize the evidence used by study design and limitations*,* Ranapurwala et al revealed several internal and external validity concerns in the opioid safety studies.^11^ It can be argued that the CDC guidelines are a work in progress, and having recommendations based on some level of evidence is better than having no recommendations at all. However, it can be questioned whether recommendations that are crucial for safe and appropriate pain treatment should be based on limited evidence. Ranapurwala et al provide recommendations to overcome concerns related to validity in future research, so that a refined version of the guidelines can have a stronger evidence base.^11^

## 4 COMPLIANCE WITH CLINICAL GUIDELINES

Aside from the level of evidence, it is useful to know to what extent these guidelines are followed in practice. In the current issue of *Pharmacoepidemiology and Drug Safety*, two papers have assessed compliance with CDC recommendations.^12^, ^13^ Hunnicutt et al have studied opioid prescribing in nursing homes in the United States in light of the recommendations to use immediate‐release opioids when starting treatment.^12^ Between 2011 and 2013, the initiation of opioid therapy in more than 182 000 long‐stay nursing home residents was largely aligned with the CDC prescribing guidelines, with only 2% of patients receiving long‐acting opioids at the start of therapy.^12^ Young et al studied the recommendation to prescribe the lowest effective dosage when initiating opioid therapy, while avoiding an increase in dosages of extended release and long‐acting (ER/LA) opioids to 90 morphine milligram equivalents (MME) or more per day, in combination with the label recommendation to establish opioid tolerance before initiation of higher dose ER/LA opioids.^13^ A large database covering over 147 million inhabitants in the United States with employer‐based insurance was used to identify adult patients initiating ER/LA opioids greater than or equal to 90MME. The results showed that 38% of the 372 038 initiators did not have evidence that opioid tolerance was established prior to initiation of greater than or equal to 90 MME of ER/LA opioids, which is not in line with label recommendations.^13^ It is unclear whether prior use of opioids paid in cash—and therefore unobserved in the database—may have contributed to the lack of evidence on established opioid tolerance. Young et al also found that nontolerant patients had a 37% increased risk of diagnosis with opioid poisoning after initiation. This increased risk was limited to the first 7 days after initiation.^13^

Compliance with clinical guidelines is an essential prerequisite for the functioning of health systems. But clinical guidelines are typically based on the average patient; there may be certain patients with individual circumstances that justify deviating from guidelines. For example, there may be patients that require treatment with an extended release formulation, or with a higher dose than 90 MME at onset of their treatment. This also applies to patients with a history or high susceptibility of opioid dependence; this group represents a particularly disadvantaged and challenging population. Providing these patients with opioids in a balanced fashion remains critical. Since this is a high‐risk population, close clinical monitoring, management of abuse risk, and adequate access to opioid dependence treatment are crucial when opioid analgesics are justifiably used for pain management.

## 5 POTENTIAL IMPACT ON THE CRISIS OF UNCONTROLLED PAIN

Although the focus on appropriate prescribing and dispensing is understandable given the current opioid abuse and misuse crisis, there is a striking absence on research related to the other crisis, ie, the lack of equal access to medically justified opioids. A review of 46 articles published between 2007 and 2013 showed that 31.8% of the patients with cancer did not receive adequate pain relief.^14^ Current national drug control systems are thought to contribute to unequal access to opioid medicines, in addition to other factors such as a lack of knowledge and education, societal attitudes, and economic issues.^15^ We need to reflect on the question whether our efforts to combat the opioid epidemic (nonmedical use crisis) have a negative impact on the crisis of uncontrolled pain.

Societal attitudes regarding the medical use of opioid analgesics may have changed because of the opioid epidemic. In discussions addressing this crisis, people may not always distinguish between overdose, misuse or illegal diversion of prescribed opioids, and use of illicit opioids. This confusion may result in a disproportionate generalized fear of opioids, limiting access for patients in medical need. Some experts believe that most patients with opioid dependence are recreational drug users who become dependent, rather than patients with pain becoming patients with opioid dependence.^10^ National drug control measures implemented to combat the crisis of nonmedical use may also potentially impact access to opioids for patients in legitimate medical need, although there is lack of solid evidence to support this theory. Likewise, it is still unclear whether the recently implemented opioid prescribing policies and prescription drug monitoring programs have a significant impact on the levels of nonmedical use of opioids in practice. Additionally, there is a need for research investigating other unintended consequences of policy measures and regulatory actions, such as the transitioning from prescription opioids to illicit opioids, including heroin. There is also a broader need for evidence to address data gaps on safety and the long‐term effectiveness of different types of pain management treatments for different types of pain, including treatment with opioid analgesics. Future research could for example focus on the magnitude of the opioid‐induced hyperalgesia effect for different kinds of patients and treatments, and critical success factors for effective long‐term opioid treatment in managing different types of pain.

Although it is beyond any doubt that nonmedical use and diversion of opioids should be battled, this should not go at the expense of balanced strategies to ensure access to medicines that are legitimately on the market for patients in need of essential pain relief. The issue is how to monitor and minimize potential unintended consequences for these patients. The Access to Opioid Medication in Europe (ATOME) project—aimed at the increase of access to opioid medicines in 12 countries with statistical evidence of low opioid consumption‐signaled clearly the importance of sustained investments in public health and education, and improved legal and regulatory systems to ensure safe and appropriate treatment of pain.^16^ Perhaps, one of the solutions for both crises lies in better education of health care professionals, patients and policymakers, in parallel with a more balanced view presented in the media.

### CONFLICT OF INTERESTS

The Division of Pharmacoepidemiology and Clinical Pharmacology of Utrecht University is designated as a WHO Collaborating Centre for Pharmaceutical and Regulation. The WHO Collaborating Centre for Pharmaceutical Policy and Regulation receives no direct funding or donations from private parties, including pharma industry. Research funding from public‐private partnerships, eg, IMI, TI Pharma (www.tipharma.nl), is accepted under the condition that no company‐specific product or company related study is conducted. The Centre has received unrestricted research funding from public sources, eg, Netherlands Organization for Health Research and Development (ZonMW), the Dutch Healthcare Insurance Board (ZIN), EU 7th Framework Program (FP7), the Dutch Medicines Evaluation Board (MEB), and the Dutch Ministry of Health, Welfare and Sport.
